# Impact of Flaxseed Oil Supplementation on Tobacco Dependence, Craving, and Haematological Parameters in Tobacco-Dependent Subjects

**DOI:** 10.7759/cureus.57101

**Published:** 2024-03-28

**Authors:** Anjali Singh, Narsingh Verma, Surya Kant, Ajay K Verma, Adarsh Tripathi, Kshitij Bhardwaj

**Affiliations:** 1 Department of Physiology, King George's Medical University, Lucknow, IND; 2 Department of Respiratory Medicine, King George's Medical University, Lucknow, IND; 3 Department of Psychiatry, King George's Medical University, Lucknow, IND

**Keywords:** cbc, tobacco cessation, tobacco craving, tobacco dependence, flaxseed, tobacco

## Abstract

Background

Tobacco is prevalently used in smoking or smokeless forms and remains a major public health concern worldwide, with its adverse effects on overall health. Omega-3 fatty acid (FA) has shown its promising effects in various health conditions.

Objective

The purpose of this study was to evaluate the effect of flaxseed oil (omega-3 supplementation) on tobacco dependence, craving, withdrawal symptoms, and haematological parameters in tobacco users.

Methods

In this randomised, single-blind, placebo-controlled study, 104 tobacco users (54 in the omega-3 group and 50 in the placebo group) were supplemented with 10 ml of food-grade flaxseed oil and 10 ml of placebo for six months, respectively. Their demographics, frequency of daily tobacco use, tobacco dependence, tobacco craving, tobacco withdrawal symptoms, and complete blood count (CBC) were assessed at baseline (before intervention) and after a six-month intervention.

Results

The demographic characteristics of the two groups were similar except for gender at baseline. There were 50 males and four females in the omega-3 group, while there were 42 males and eight females in the placebo group. After a six-month flaxseed oil intervention, BMI values showed a significant reduction (p = 0.0081) in the omega-3 group when compared to baseline; however, CBC parameters did not show any significant changes when comparing baseline to follow-up values. On the contrary, haemoglobin and red blood cells (RBCs) showed significant changes when comparing the follow-ups of the omega-3 group with the placebo group, indicating p = 0.0016 and p = 0.0163, respectively. Also, omega-3 effectively decreased daily tobacco use frequency (p<0.0001), tobacco dependence (p<0.0001), and craving (p<0.0001).

Conclusion

Supplementation of 10 ml of flaxseed oil per day (omega-3 FA) for six months significantly reduced tobacco dependence and cravings. Additionally, the flaxseed oil supplementation effectively reduced the frequency of daily tobacco intake and modulated tobacco withdrawal symptoms. Thus, our results suggest that flaxseed oil supplementation is a useful adjunct for tobacco users who intend to quit tobacco use.

## Introduction

Tobacco use remains a leading cause of preventable death worldwide, contributing to a myriad of health complications, including neurodegenerative disease, cardiovascular disease, respiratory disorders, cancer, etc. [1]. The fact behind this is that nicotine addiction is a formidable barrier to tobacco cessation efforts [2]. Despite the availability of various pharmacological and behavioural interventions [3-5], many individuals struggle to overcome strong tobacco urges. The reason underlying this is the nicotine addiction associated with neuroinflammation, oxidative stress, and dysregulation of neurotransmitter systems, including dopaminergic and serotonergic neurotransmission [6]. Some other factors aligning with tobacco use include psychological factors (such as stress, anxiety, negative emotions, curiosity, etc.), social and environmental factors (such as peer pressure, family environment, etc.), marketing and advertising (misleading advertisements of tobacco products), accessibility, and availability (ease of access in any location with affordable purchase prices) [7].

Emerging evidence suggests that omega-3 fatty acid (FA) supplementation has been shown to mitigate synaptic plasticity and neurobiological mechanisms in the brain influencing dopamine and serotonin pathways. These pathways are particularly associated with the release of dopamine and serotonin, which are neurotransmitters for feelings of pleasure and happiness and thus implicated in reward processing and dependence [8]. These effects may contribute to the attenuation of tobacco craving and withdrawal symptoms (anger, anxiety, depression, difficulty concentrating, weight gain, insomnia, restlessness, etc.) and, subsequently, tobacco cessation [9]. Furthermore, omega-3 FAs have been shown to regulate mood and cognitive function, factors that play a significant role in smoking behaviour and relapse prevention. Studies have suggested that omega-3 supplementation may improve mood, reduce stress, and enhance cognitive function, potentially addressing some of the psychological barriers to quitting smoking [10].

Omega-3 FAs, particularly eicosapentaenoic acid (EPA) and docosahexaenoic acid (DHA) exert a range of beneficial effects on human health, including anti-inflammatory, anti-oxidative, cardiovascular, and neuroprotective properties. Omega-3 FAs are found abundantly in fish oil, flaxseed oil, and other sources [11,12]. Albeit flaxseed oil, rich in omega-3 FA, particularly alpha-linolenic acid (ALA), derived from the seeds of the flax plant (*Linum usitatissimum*), has garnered attention for its diverse bioactive constituents and potential health benefits [13].

Additionally, omega-3 FA supplementation has been linked to modulation of immune function, improvement in vascular health, and regulation of platelet activity, all of which can lead to alterations in markers of physiological health, such as complete blood count (CBC) parameters. [14] While the mechanisms underlying these effects are not fully elucidated, potential explanations include the anti-inflammatory and vasodilatory properties of omega-3 FAs, which may influence haematological parameters such as red blood cell (RBC) count, white blood cell (WBC) count, and platelet function. [15]

Most research on tobacco dependence, craving, and withdrawal among smokers and smokeless tobacco users has been reported with the administration of EPA, DHA, fish oil, and pharmacological treatment. [3-5,15-19] However, to the best of our knowledge, no study has attempted to determine the effect of flaxseed oil (omega-3 FA) among tobacco users; therefore, this study was conducted based on the hypothesis that flaxseed oil supplementation may reduce tobacco craving and positively influence CBC parameters. Thus, the current study aims to evaluate the effect of flaxseed oil (omega-3 supplementation) on tobacco dependence, craving, withdrawal symptoms, and haematological parameters in tobacco users.

## Materials and methods

This randomised, single-blind, placebo-controlled study was performed at King George’s Medical University, Lucknow, Uttar Pradesh, India. The institutional ethics committee approved this study (reference number 102th ECM II B-Ph.D/P2, dated June 2, 2021). A total of 200 regular tobacco users were screened, followed by 157 enrollments randomized (using the computer-generated random number table method) to two groups, i.e., 78 in the omega-3 group and 79 in the placebo group. In the omega-3 group, only 54 subjects gave their follow-up, and 24 subjects were lost to follow-up, whereas in the placebo group, 50 subjects gave their follow-up, and 29 subjects were lost to follow-up. Tobacco dependence was confirmed by the International Classification of Diseases, Tenth Revision (ICD-10) criterion [16]. As per the inclusion criteria, subjects attending the tobacco cessation clinic of the institute aged 18-60 years, either male or female, and those who volunteered for tobacco cessation were recruited. We excluded pregnant women, subjects with any known comorbidity (such as lung diseases, cardiac illness, diabetes, cancer, psychiatric disorders, or human immunodeficiency virus (HIV)), subjects on any kind of medication, subjects on nicotine replacement therapy for tobacco cessation, subjects associated with any other trial, known alcoholics, and subjects already on omega-3-rich supplementation or those who were taking flaxseed in their diet.

Baseline demographic characteristics were collected from all recruited subjects (age, gender, marital status, tobacco use, age at the beginning of tobacco use, years of tobacco use, awareness about omega-3 FA, weight, height, body mass index (BMI), waist-hip ratio (WHR), and frequency of daily tobacco use). Additionally, we measured clinical parameters using validated questionnaires. Nicotine dependence was assessed using the Fagerström Test for Nicotine Dependence (FTND) [17] and the Fagerström Test for Nicotine Dependence-Smokeless Tobacco (FTND-ST). [18] The FTND and FTND-ST contain six questions each. The responses to all the questions were added up to yield a total score (total score >5 = significant dependence; total score <4 = low or moderate dependence). Tobacco craving was assessed using the 10-item Questionnaire on Smoking Urges (QSU-Brief) [19] and the 10-item Questionnaire on Smokeless Tobacco Urges (Modified Questionnaire for Smokeless Tobacco from QSU-Brief (QSU-Brief (Modified))) upon the subject’s enrollment in the study. The QSU-Brief contains 10 items whose scores are summed up to yield a total QSU score. We also assessed tobacco withdrawal symptoms using the Minnesota Nicotine Withdrawal Scale (MNWS). The MNWS has subscales, each with five options underlying respective scores of 0-four (0 = none; 1 = slight; 2 = mild; 3 = moderate; 4 = severe). Furthermore, 5 ml of blood was collected from all the study subjects. A CBC was performed using lyse and diluent in a Medonic automated haematology analyzer (Boule Medical AB, Stockholm, Sweden).

**Figure 1:**
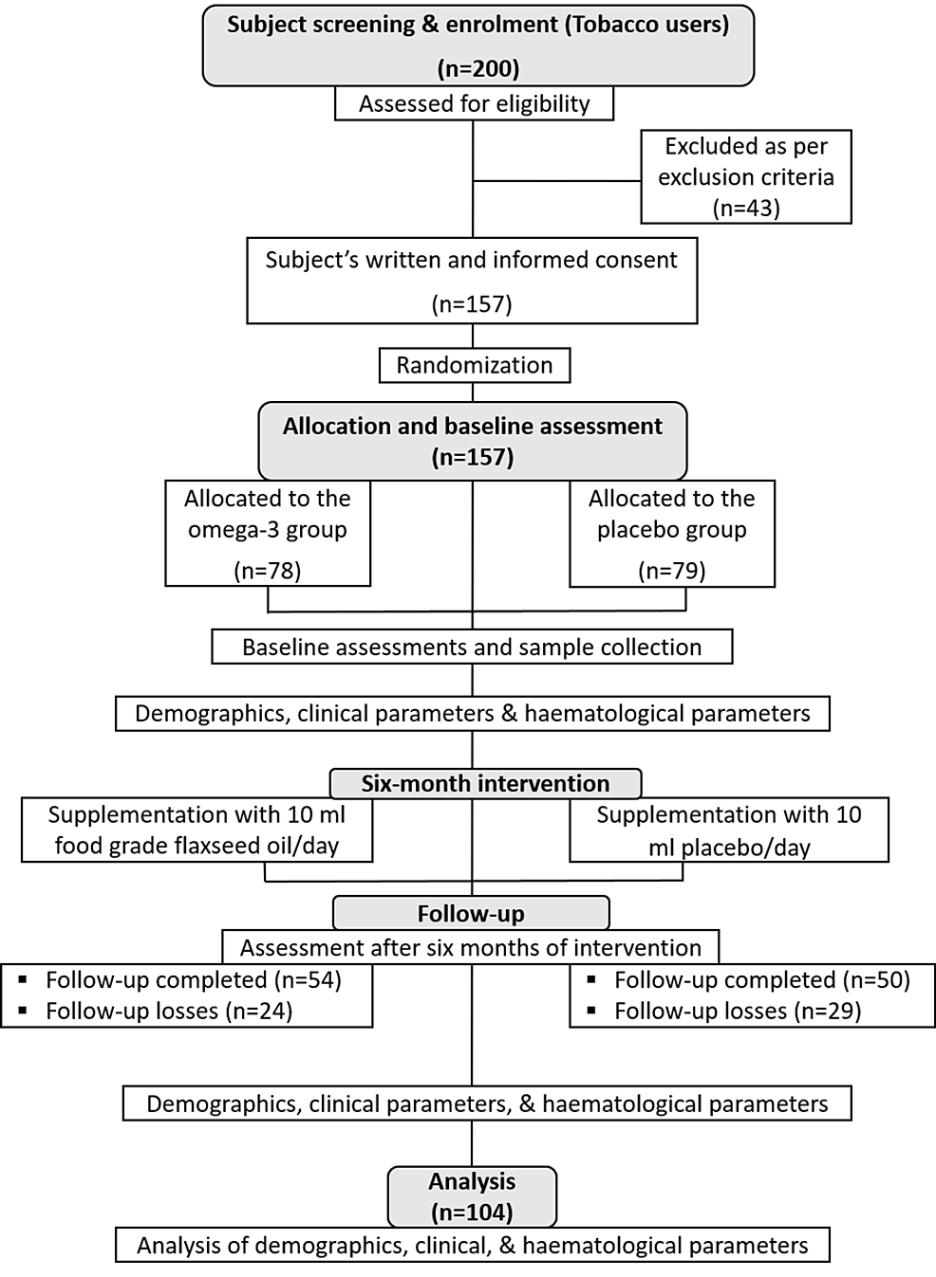
Study flowchart

After the baseline assessments, subjects were instructed to consume 10 ml/day of food-grade pure flaxseed oil (procured from Ceyon Healthcare India Private Limited, Lucknow, India) in the omega-3 group and 10 ml/day mustard oil (procured from Shudh Bharat, a unit of Ceyon Healthcare India Private Limited) in the placebo group for six months. To maintain the blindness of the study, supplements were provided to patients in identical black glass bottles in both groups labelled with a code (understandable only to the investigator and not by the study subject). Each bottle contained a 10 ml dose for a day. We also monitored compliance through regular message reminders and telephonic calls every week. The subject attendants were also communicated with to ensure the subject’s adherence to supplementation. Both group subjects were asked to return empty supplement bottles after monthly consumption and collect supplement bottles for the next month. The study flow chart is shown in Figure 1.

Enrolled patients were followed up after six months of their enrollment, along with six-month supplementation. Thereafter, their clinical and biochemical assessments were repeated.

Written and informed consent was obtained from all the subjects enrolled in the study. In addition to this, the study was done according to the Declaration of Helsinki. The study was registered with the Clinical Trial Registry-India at www.ctri.nic.in. (reference number: CTRI/2022/02/040681).

Statistical analysis

All data were presented as the mean, standard deviation (SD), number (n), and percentage (%). GraphPad Prism 5 (GraphPad Software, La Jolla, CA) was used to sum up the data. The data were checked for normality. Discrete variables were compared between groups using the chi-square test. The differences between the two groups were assessed using the Mann-Whitney U test and the unpaired t-test. For differences within the groups, the Wilcoxon matched pair test and the paired t-test were used. The p-values were considered significant at p <0.05.

## Results

The average age of the omega-3 group (47.02±10.96 years) and of the placebo group (48.48±9.22 years) was found to be non-significant (p = 0.4653). There were 92.59% males and 7.41% females in the omega-3 group, while there were 84% males and 16% females in the placebo group. The baseline demographic characteristics and clinical parameters of the study subjects are shown in Table 1.

**Table 1:** Baseline demographic characteristics and clinical parameters of the study subjects Data are expressed in mean±SD, and n(%); ^†^unpaired t-test; ^$^chi-square test; ^₸^Mann-Whitney U test, ^ns^p>0.05 n: number of subjects; %: percentage; SD: standard deviation; FA: fatty acid; BMI: body mass index; WHR: waist-hip ratio; FTND: Fagerström Test for Nicotine Dependence; FTND-ST: Fagerström Test for Nicotine Dependence-Smokeless Tobacco; QSU-Brief: Questionnaire on Smoking/Smokeless Urges

Demographic characteristics and clinical parameters of both groups		Omega-3 (n=54)	Placebo (n=50)	Significance
Age (years)		47.02±10.96	48.48±9.22	^†^p^ns^=0.4653
Gender	Male	50 (92.59%)	42 (84%)	^$^p^ns^=0.539
	Female	4 (7.41%)	8 (16%)	
Marital status	Married	52 (96.30%)	47 (94%)	^$^p^ns^=0.857
	Unmarried (single)	2 (3.70%)	3 (6%)	
Tobacco users	Smokers	16 (29.63%)	15 (30%)	^$^p^ns^=0.099
	Smokeless tobacco users	38 (70.37%)	35 (70%)	
Age at the beginning of tobacco use (years)		26.89±12.22	28.98±11.39	^₸^p^ns^=0.1804
Years of tobacco use		21.10±12.83	20.22±10.25	^†^p^ns^=0.7008
Awareness about omega-3 FA	Yes	8 (14.81%)	16 (32%)	^$^p^ns^=0.613
	No	46 (85.19%)	34 (68%)	
Weight (kg)		65.06±13.59	69.68±11.25	^₸^p^ns^=0.0842
Height (cm)		166±8.40	164.2±8.83	^₸^p^ns^=0.2463
BMI		23.62±4.87	25.37±3.69	^†^p^ns^=0.0516
WHR		0.97±0.06	0.97±0.09	^₸^p^ns^=0.0885
Frequency of daily tobacco use		8.63±5.70	6.92±3.85	^₸^p^ns^=0.1511
Tobacco dependence (FTND-ST & FTND score)		6.33±2.36	5.78±2.08	^₸^p^ns^=0.0668
Tobacco craving (QSU-Brief score)		50.26±9.43	49.98±7.91	^₸^p^ns^=0.4757

Baseline demographic characteristics and clinical parameters

The demographics of age, gender, marital status, tobacco users, age at the beginning of tobacco use, years of tobacco use, awareness of omega-3 FA, weight, height, BMI, WHR, frequency of tobacco use, FTND scores, and QSU scores were found to be non-significant when compared in both groups at baseline (before intervention).

Changes from baseline to follow-up in demographic characteristics after the six-month intervention

In the omega-3 group, the values of BMI showed a significant decrease (p = 0.0081) when comparing baseline to follow-up. Furthermore, BMI and WHR showed significant changes when comparing the follow-ups of both groups, with p = 0.0002 and p = 0.026, respectively (Table 2).

**Table 2:** Changes from baseline to follow-up in demographic characteristics within the group and comparison of changes between the two groups Data are expressed in mean±SD; ^I^comparison between the mean baseline and follow-up of omega-3 group; ^II^comparison between the mean baseline and follow-up of placebo group; ^III^comparison between the follow-ups of the two groups; ^#^Wilcoxon matched pair test; ^¥^paired t-test; ^₸^Mann-Whitney U test; ^†^unpaired t-test; ^ns^p>0.05; *p≤0.05; **p≤0.01; ***p≤0.001 n: number of subjects; SD: standard deviation; BMI: body mass index; WHR: waist-hip ratio

Demographics	Omega-3 baseline (n=54)	Omega-3 follow-up (n=54)	Placebo baseline (n=50)	Placebo follow-up (n=50)	p^I^	p^II^	p^III^
	Mean±SD	Mean±SD	Mean±SD	Mean±SD			
BMI	23.62±4.87	23.29±4.66	25.37±3.69	26.64±4.02	^¥^p^**^=0.0081	^¥^p^ns^=0.115	^†^p^***^=0.0002
WHR	0.97±0.06	0.97±0.05	0.97±0.09	0.99±0.06	^¥^p^ns^=0.8521	^#^p^ns^=0.0551	^₸^p^*^=0.026

Changes from baseline to follow-up in haematological parameters after the six-month intervention

The mean CBC values were non-significant at baseline among both groups. All the CBC parameters showed a slight change from baseline to follow-up in both groups, but the change was not found to be significant. On the other hand, haemoglobin (p = 0.0016), RBC (p = 0.0163), mean corpuscular haemoglobin concentration (MCHC) (p = 0.0335) and monocyte (p = 0.0353) showed significant changes when comparing the follow-ups of both groups (Table 3).

**Table 3:** Changes from baseline to follow-up in haematological parameters within the group and comparison of changes between the two groups Data are expressed in mean±SD; ^I^comparison between the mean baseline and follow-up of omega-3 group; ^II^comparison between the mean baseline and follow-up of the placebo group; ^III^comparison between the follow-ups of the two groups; ^#^Wilcoxon matched pair test; ^¥^paired t-test; ^₸^Mann-Whitney U test; ^†^unpaired t-test; ^ns^p>0.05; *p≤0.05; **p≤0.01 n: number of subjects; SD: standard deviation; CBC: complete blood count; Hb: haemoglobin; HCT: haematocrit; RBC: red blood cell; MCV: mean corpuscular volume; MCH: mean corpuscular haemoglobin; MCHC: mean corpuscular haemoglobin concentration; WBC: white blood cell count; MPV: mean platelet volume

CBC		Omega-3 baseline (n=54)	Omega-3 follow-up (n=54)	Placebo baseline (n=50)	Placebo follow-up (n=50)	p^I^	p^II^	p^III^
		Mean±SD	Mean±SD	Mean±SD	Mean±SD			
Hb (gm/dL)		13.32±1.48	13.77±1.83	12.49±1.98	12.48±2.10	^#^p^ns^=0.6923	^¥^p^ns^=0.3896	^₸^p^**^=0.0016
HCT (%)		39.26±4.68	41±5.45	36.65±8.52	38.39±6.79	^¥^p^ns^=0.2117	^#^p^ns^=0.2046	^†^p^ns^=0.0595
RBC count (million cells/uL)		4.61±0.43	4.61±0.58	4.36±0.71	4.24±0.77	^¥^p^ns^=0.7025	^¥^p^ns^=0.8479	^†^p^*^=0.0163
MCV (fL)		86.32±14.33	88.52±7.84	87.06±9.85	90.04±8.26	^#^p^ns^=0.7117	^#^p^ns^=0.174	^₸^p^ns^=0.9449
MCH (pg/cell)		29.44±3.27	29.9±2.94	28.57±3.54	28.92±2.2	^#^p^ns^=0.323	^¥^p^ns^=0.3026	^₸^p^ns^=0.1719
MCHC (gm/dL)		33.15±1.75	33.83±2.37	32.21±2.71	32.61±2.06	^#^p^ns^=0.4049	^#^p^ns^=0.2701	^₸^p^*^=0.0335
WBC count (thou/uL)		7.95±2.60	7.99±2.42	7.97±3.46	7.79±3.08	^#^p^ns^=0.33	^¥^p^ns^=0.6667	^†^p^ns^=0.7424
Differential WBC count	Neutrophils (%)	61.62±10.41	62.15±11.74	61.74±14.22	61.71±4.42	^¥^p^ns^=0.5271	^#^p^ns^=0.3632	^₸^p^ns^=0.7006
	Lymphocyte (%)	29.25±9.26	29.47±10.41	28.22±10.06	28.02±7.47	^¥^p^ns^=0.6931	^#^p^ns^=0.4894	^₸^p^ns^=0.6834
	Eosinophils (%)	3.38±1.91	3.56±2.74	3.69±2.13	3.72±1.78	^#^p^ns^=0.5177	^#^p^ns^=0.2655	^₸^p^ns^=0.3659
	Monocyte (%)	2.79±2.06	2.91±2.86	3.77±2.92	3.61±2.3	^#^p^ns^=0.8239	^#^p^ns^=0.2447	^₸^p^*^=0.0353
	Basophils (%)	0.12±0.3	0.17±0.38	0.19±0.52	0.16±0.27	^#^p^ns^=0.3997	^#^p^ns^=1	^₸^p^ns^=0.5336
Platelets count (thou/uL)		176.6±63.98	177.2±65.63	156±52.94	154.6±82.47	^¥^p^ns^=0.725	^#^p^ns^=0.3871	^₸^p^ns^=0.1351
MPV (fL)		11.57±1.76	12.37±1.53	11.62±2.06	11.56±1.72	^¥^p^ns^=0.2426	^#^p^ns^=0.0523	^₸^p^ns^=0.2064

Changes from baseline to follow-up in frequency of daily tobacco use after a six-month intervention

**Figure 2:**
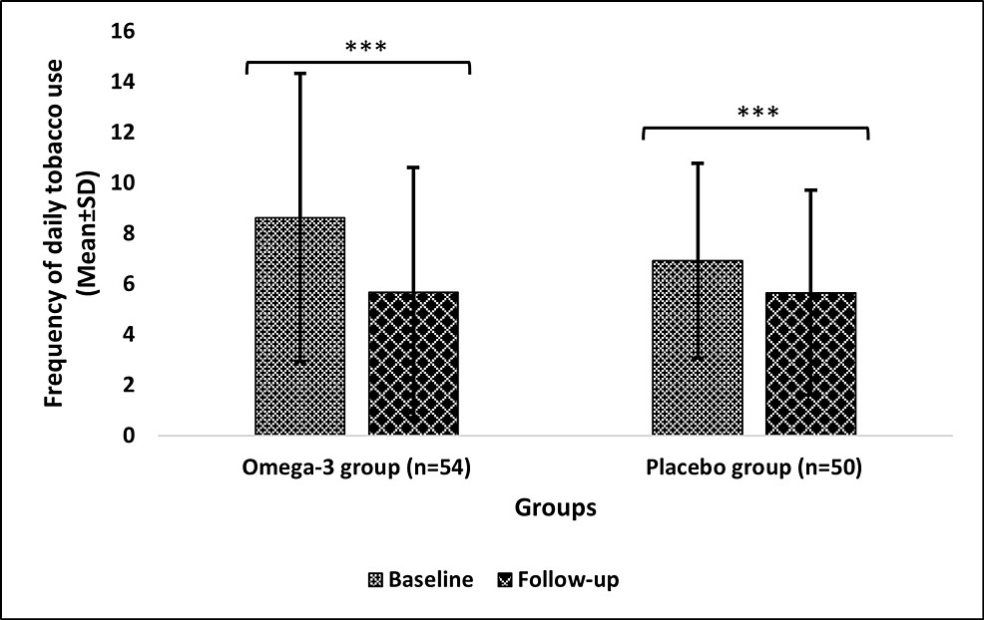
Frequency of daily tobacco use of the omega-3 and placebo groups Data are expressed in mean±SD; level of significance: ***p≤0.001 n: number of subjects; SD; standard deviation

The frequency of daily tobacco use significantly decreased (p<0.0001) from 8.63±5.70 at baseline to 5.67±4.95 after the follow-up in the omega-3 group. However, the placebo group showed a slight decrease from 6.92±3.85 (at baseline) to 5.66±4.05 (after follow-up), but still with a significant change (p<0.0001) (Figure 2).

Changes from baseline to follow-up in tobacco dependence after the six-month intervention

**Figure 3:**
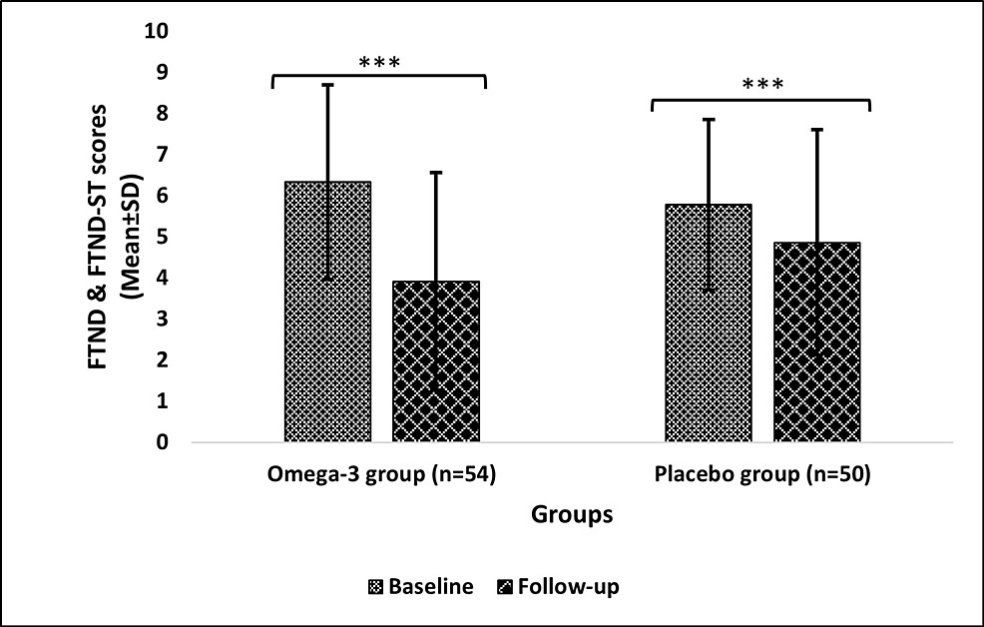
Tobacco dependence (FTND scores) of the omega-3 and placebo groups Data are expressed in mean±SD; level of significance: ***p≤0.001 n: number of subjects; SD: standard deviation; FTND: Fagerström Test for Nicotine Dependence; FTND-ST: Fagerström Test for Nicotine Dependence

The FTND scores showed significant change (p<0.0001) in both groups when comparing baseline to follow-up. However, the change in the omega-3 group was from 6.33±2.36 at baseline to 3.91±2.65 after the follow-up. While in the placebo group, there was a slight decrease from 5.78±2.08 at baseline to 4.86±2.74 after the follow-up (Figure 3).

Changes from baseline to follow-up in tobacco craving after the six-month intervention

**Figure 4:**
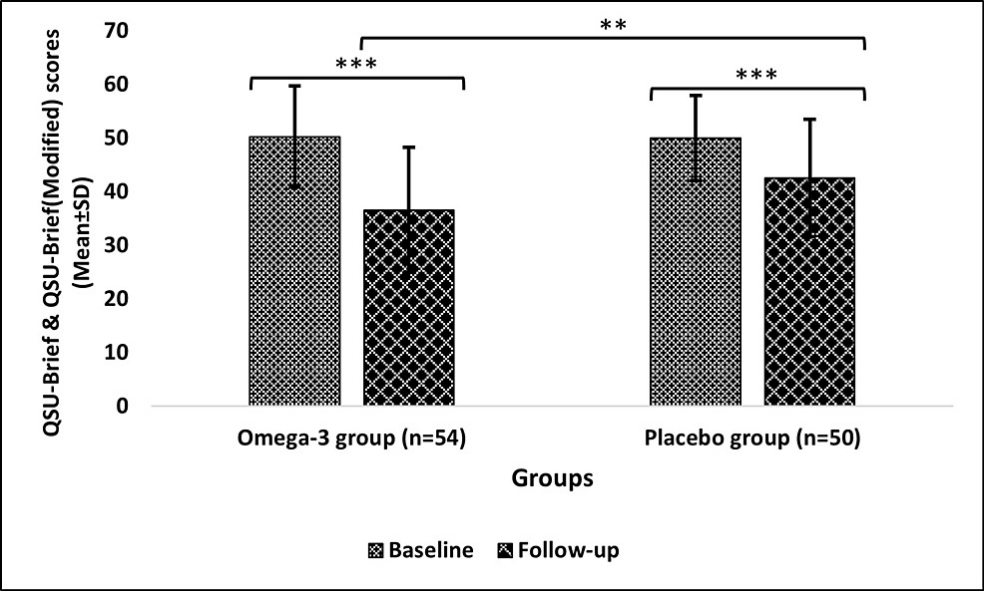
Tobacco craving (QSU scores) of the omega-3 and placebo groups Data are expressed in mean±SD; level of significance: **p≤0.01, ***p≤0.001 n: number of subjects; SD: standard deviation; QSU-Brief: 10 item-Questionnaire on Smoking Urges; QSU-Brief (Modified): 10 item-Questionnaire on Smokeless Tobacco Urges

The QSU not only showed significant change (p<0.0001) in both groups on comparing baseline to follow-up but also on comparing follow-ups of both groups with p = 0.0085 (Figure 4).

Changes from baseline to follow-up in tobacco withdrawal symptoms after the six-month intervention

**Figure 5:**
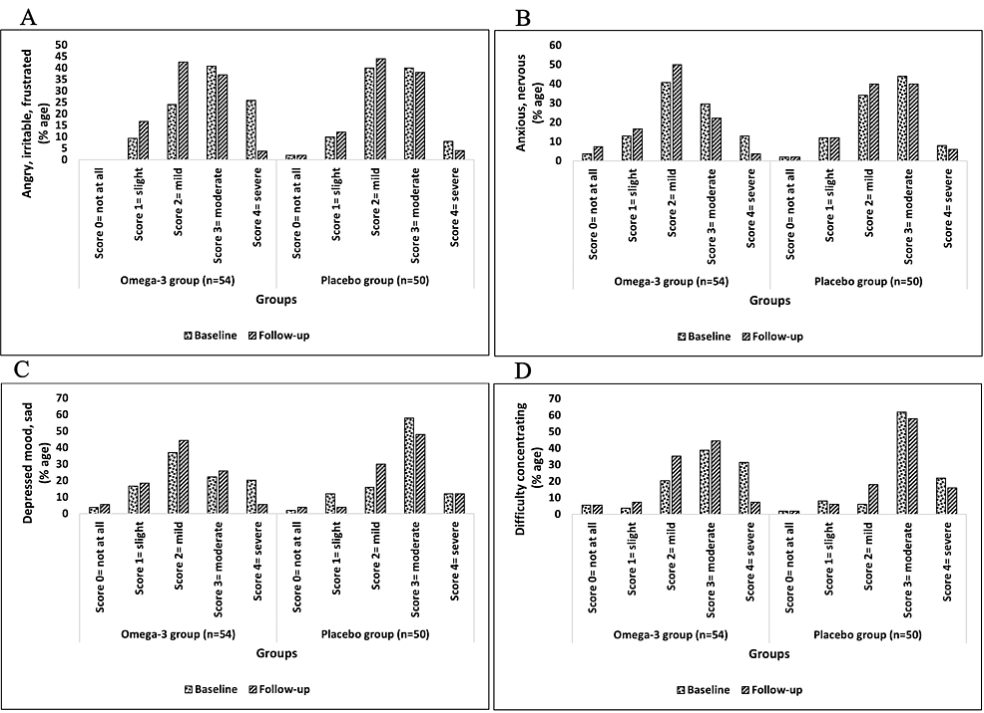
The MNWS subscales (tobacco withdrawal symptoms) of omega-3 and placebo groups (part I) This figure represents four withdrawal symptoms along with the change in the scores (percentage) of each withdrawal symptom under MNWS of both groups at baseline and follow-up, respectively. n: number of subjects; %age: percentage; MNWS: Minnesota Nicotine Withdrawal Scale A: Angry, irritable, frustrated; B: Anxious, nervous; C: Depressed mood, sad; D: Difficulty concentrating

**Figure 6:**
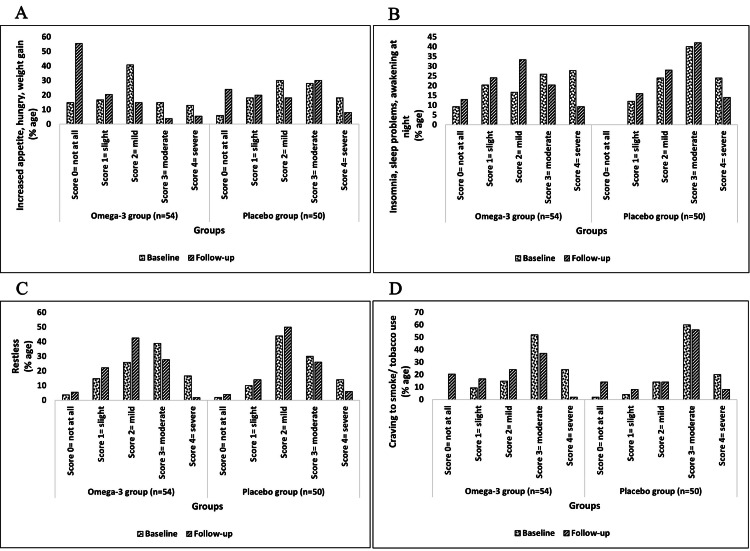
MNWS subscales (tobacco withdrawal symptoms) of omega-3 and placebo groups (part II) This figure represents four withdrawal symptoms along with the change in the scores (percentage) of each withdrawal symptom under MNWS of both groups at baseline and follow-up respectively n: number of subjects; %age: percentage; MNWS: Minnesota Nicotine Withdrawal Scale A: Increased appetite, hunger, weight gain; B: Insomnia, sleep problems, awakening at night; C: Restless; D: Craving to smoke/ tobacco use

Among the withdrawal symptoms measured on the MNWS scale, the severity (score of 4) of anger (25.93%), anxiousness (12.96%), depression (20.37%), difficulty in concentrating (31.48%), increase in appetite (12.96%), insomnia (27.78%), restlessness (16.67%), and craving to smoke or use tobacco (24.07%) at baseline was notably reduced to 3.7%, 3.7%, 5.56%, 7.41%, 5.56%, 9.26%, 1.85%, and 1.85%, respectively, after follow-up in the omega-3 group. However, in the placebo group, the severity of some symptoms was slightly reduced, and a few symptoms remained at the same stage when comparing baseline to follow-up (Figures 5-6).

## Discussion

Several studies have investigated the effects on tobacco craving and smoking behaviour using pharmacological approaches (bupropion sustained release (SR), varenicline, etc.) as well as non-pharmacological approaches (omega-3 FA supplementation in the form of EPA, DHA, and fish oil). However, non-pharmacological approaches are limited to the Western world, whereas pharmacological approaches have been reported in the Indian scenario. In addition, the effects of omega-3 FA (ALA)-rich flaxseed oil have rarely been focused on craving reduction, withdrawal symptoms, or tobacco cessation outcomes. Therefore, research is needed to study the effect of omega-3 supplementation (ALA) in the form of flaxseed oil on tobacco users, as the available literature focuses on EPA and DHA (omega-3).

The present randomised, single-blind, placebo-controlled study among tobacco-dependent subjects revealed a significant change (p = 0.0002) in the BMI in omega-3 (flaxseed oil) vs. placebo group follow-ups. Consistently, the other study also resulted in a significant change in BMI when comparing the follow-ups of the omega-3 (fish oil) group with the placebo (mineral oil) group among smokers [20]. This is because omega-3 boosts metabolism, which burns fat and stimulates leptin, which directs the brain to lower the activity of neuropeptide Y (a neurotransmitter) that suppresses appetite and causes people to eat less; hence, lowering their body weight and BMI [21].

Moreover, in a study, 2.4 g/day of EPA in combination with 1.2 g/day of DHA was administered among smokers for six months, reporting a significant reduction in granulocytes and an increase in lymphocytes [22]. On a contrary note, our study in the omega-3 group found a slight increase in both granulocytes (neutrophils, eosinophils, and basophils) and lymphocytes but no significant change. A high granulocyte/lymphocyte ratio has been linked to inflammation [23] and an increased risk of cancer [24], according to available evidence implying the potential of omega-3 to lower the risk of developing lung cancer. Additionally, a previous interventional (12-week) study with daily omega-3 FA supplementation gel capsules (fish oil) and placebo gel capsules (sunflower oil) along with nutrition counselling showed increased haemoglobin levels in the omega-3 group but without significance [25]. In line with this finding, our result also showed a slight increase in the omega-3 group but did not reach the level of significance.

Besides this, it is already known that omega-3 intake and smoking are inversely related [26]. Moving forward in the same context, the results of a study stated that the omega-3 FA group showed a greater reduction in daily cravings for cigarettes and cigarettes smoked per day than the placebo group. This difference increased from the baseline to the three-month follow-up [27]. This inference corroborates the findings of our study, which showed a considerable drop in the omega-3 group's daily tobacco use frequency. On the other hand, in a different study, low-dose DHA supplementation administered to a specific sample of smokers for a short period without a control group failed to reduce the number of cigarettes smoked over the course of the study period [28]. In contrast to this research, our study found a significant cutback in the frequency of tobacco use.

Furthermore, another study reported a significant reduction in nicotine dependence level (FTND scores) during the 90-day treatment of smokers with fish oil capsules (omega group) in comparison to mineral oil capsules (placebo group) [20]. Similar to this finding, our study also reported a significant decrease in nicotine levels in the omega-3 and placebo groups. The findings of our study are in accordance with previous research suggesting that omega-3 FAs are effective in significantly reducing the levels of nicotine dependence, as also observed by Sadeghi-Ardekani et al. (2018) [27]. More intriguingly, according to the findings of a pilot study, daily administration of EPA (2,710 mg) and DHA (2,040 mg) for a month deemed a significant decrease in tobacco craving (QSU scores) from baseline to follow-up among regular smokers compared to placebo (mineral+soybean oil capsule) treatment [29]. Likewise, our findings also demonstrate a significant reduction in QSU scores, implying a decrease in tobacco cravings in both omega-3 and placebo groups. This reduction in the frequency of tobacco use, its dependence, and subsequently craving is due to the presence of sufficient concentrations of omega-3 FA, which account for proper neurotransmission, leading to the smooth functioning of the dopaminergic pathway (alpha-4 beta-2 (α4β2) nicotinic acetylcholine receptor), stimulating adequate amounts of dopamine release even in the absence of nicotine intake [30-32]. Moreover, by integrating information from the Kyoto Encyclopaedia of Genes and Genomes (KEGG) pathway, more light can be shed on changes in neurotransmission as well as the structure and function of α4β2 in omega-3 FA supplementation [33]. A possible explanation for the significant changes in the placebo group is the placebo effect. On top of these findings, there was also a notable change in tobacco withdrawal symptoms (MNWS) in both groups.

Simultaneously, studies with different approaches, i.e., the use of various pharmaceuticals, including bupropion SR [3], varenicline [4], and buprenorphine-naloxone maintenance therapy [5], have demonstrated their effectiveness in reducing cravings for tobacco, tobacco dependence, and daily tobacco use, respectively. In contrast, our study focused on the use of dietary supplements (flaxseed oil) without the use of pharmaceutical intervention, and the findings showed a substantial difference in the omega-3 group in terms of frequency of tobacco use per day, tobacco dependency, and craving.

As a result of our approach, we believe that flaxseed oil supplementation reduces tobacco dependence and craving. However, the result of the placebo treatment reducing the severity of tobacco withdrawal symptoms remains contradictory. Our study had a few limitations. It followed a single-blind study design with small sample size and only two study time points, with males constituting the bulk of study subjects and comorbidities excluded.

## Conclusions

Our findings suggest that 10 ml of flaxseed oil taken daily for six months is potentially effective in reducing the frequency of daily tobacco use, tobacco dependence, and craving, and modulating its withdrawal symptoms, implying the potent role of omega-3 FA supplementation in aiding tobacco cessation. However, the role of omega-6 FA should not be overlooked, as a balanced ratio of omega-6 to omega-3 FA may optimise the effectiveness of omega-3 FA supplementation among tobacco users. Moreover, for future inferences, studies could be performed on tobacco users coupled with any communicable or non-communicable disease to identify and confirm its beneficial effects on these patients too.
